# Effects of Dietary Paper Mulberry *(Broussonetia papyrifera)* on Growth Performance and Muscle Quality of Grass Carp (*Ctenopharyngodon idella*)

**DOI:** 10.3390/ani11061655

**Published:** 2021-06-02

**Authors:** Tao Tang, Jinhai Bai, Zhipeng Ao, Zehong Wei, Yi Hu, Shaojun Liu

**Affiliations:** 1 State Key Laboratory of Developmental Biology of Freshwater Fish, College of Life Sciences, Hunan Normal University, Changsha 410081, China; tangtao9094@163.com (T.T.); b13807253562@outlook.com (J.B.); 15170482247@139.com (Z.A.); lsj@hunnu.edu.cn (S.L.); 2 Hunan Engineering Research Center for Utilization of Characteristics of Aquatic Resources, Hunan Agricultural University, Changsha 410128, China; huyi740322@163.com

**Keywords:** paper mulberry, grass carp, growth, flesh quality, myogenic regulatory factors

## Abstract

**Simple Summary:**

The quality of muscle plays an important role in improving the economic benefit of aquatic products. The paper mulberry (*Broussonetia papyrifera*, BP) grows wildly in the south of Asia. In this experiment, grass carps (initial weight: 50.0 ± 0.5 g) were fed diets with the addition of 0%, 5%, 10%, 15% and 20% BP in a control diet (crude protein: 31%, crude fat: 3.8%) for 8 weeks. In conclusion, adding 5% BP did not affect the growth of grass carp. However, the supplementation of 10% BP could improve muscle quality through improving muscle hardness, reducing fat accumulation and muscle fiber diameter, at the cost of reducing growth performance.

**Abstract:**

The present study investigated the effects of dietary paper mulberry (*Broussonetia Papyrifera*, BP) on growth performance, muscle quality and muscle growth-related mRNA expressions of grass carp. Fish (initial weight: 50.0 ± 0.5 g) were fed diets supplemented with 0% (control diet), 5%, 10%, 15% and 20% BP for 8 weeks. The results showed that increasing levels of paper mulberry linearly and quadratically decreased the special gain rate (SGR) and increased the feed conversion rate (FCR) of grass carp (*p <* 0.05). Significantly positive quadratic trends were found between paper mulberry levels and muscle crude fat or crude protein of grass carp (*p <* 0.05). In comparison to the control diet, the 10%BP and 15%BP groups had significantly decreased muscle crude fat and increased crude protein (*p <* 0.05). The levels of paper mulberry resulted in a linear and quadratic increase in water loss of grass carp muscle (*p <* 0.05), and all groups with paper mulberry supplementation were significantly higher than the control group (*p <* 0.05). Significant positive linear and quadratic trends were found between the paper mulberry levels and muscle fiber diameter or density of grass carp (*p <* 0.05). In comparison to the control diet, the significant differences were found in the 15%BP and 20%BP groups (*p <* 0.05). The muscle adhesiveness and hardness linearly and quadratically increased with the increasing levels of paper mulberry (*p <* 0.05), and both of which increased significantly when the level of paper mulberry reached 10% (*p <* 0.05). In addition, the increase in paper mulberry linearly and quadratically improved the expressions of myoblast determination protein (MyoD), myogenin (MyoG), paired box protein 7 (Pax7) and myostatin 1 (MSTN1) (*p <* 0.05). When the supplementation of paper mulberry reached 15%, the expressions of all these mRNAs were significantly higher than those of the control group (*p <* 0.05). In summary, adding 5% paper mulberry did not affect the growth of grass carp. However, the supplementation of 10% paper mulberry could improve muscle quality through improving muscle hardness, reducing fat accumulation and muscle fiber diameter, at the cost of reducing growth performance.

## 1. Introduction

Paper mulberry (*Broussonetia papyrifera*) belongs to the genus Broussonetia of the mulberry family, which grows widely in the east and south of Asia, such as China and India [1]. Paper mulberry has a long history of utilization in papermaking and medicine industries [2]. Moreover, it has been widely used in feeding sheep, cows, pigs and so on. The leaves of paper mulberry contain 21.6% of crude protein and 4.3% of ether extract, both of which are higher than that of other forages [3].

It was reported that adding the appropriate content of paper mulberry in diets improved the growth performance of sheep [4], beef cattle [5] and growing rabbits [3]. However, the anti-nutrition factor tannin limited the addition of paper mulberry in livestock. Previous studies proved that tannin acid formed complexes with carbohydrates and proteins, resulting in a decrease in feed utilization and growth performance of animals [6,7]. Yang et al. [8] found that adding more than 10% of paper mulberry damaged the apparent digestibility of proteins and dry matter of pigs. Moreover, the high content of fiber affected the monogastric animals’ utilization of paper mulberry in comparison to the addition of paper mulberry in ruminants, which in sows and rabbits was lower [9,10].

There were other researchers who showed that paper mulberry improved the meat quality of livestock [11,12]. Hua et al. [11] found that dietary paper mulberry supplementation increased muscle pH, the meat color and water-holding capacity (WHC) of goat. Muscle pH has a great impact on tenderness and WHC of muscle [13]. WHC reflects the ability of muscle to prevent water from leaking out, so it is an important parameter for evaluating meat quality. The decrease in WHC not only increases the drip loss but also damages the texture of raw meat [14,15]. Xiong et al. [16] found that adding paper mulberry effectively reduced the drip loss (the amount of fluid loss from the muscle, and it is negative related to the WHC) of broiler. In addition, Yang et al. [8] found that adding 10% paper mulberry increased the contents of fat and sodium glutamate in pig muscle. Both fat and sodium glutamate are helpful in improving the flavor of meat [17]. However, in comparison to livestock, studies on the effects of dietary paper mulberry on aquatic animals are relatively scarce. Chen et al. [18] found that adding a 0.4% mixture of fermented mugwort and paper mulberry leaves improved the immunity of carp (*Cyprinus carpio*).

Different from mammals, fish have a long-lasting ability to recruit new skeletal muscle fibers [19]. A previous study proved that there was a negative correlation between the fiber diameter and hardness in the muscle of pigs [20]. Therefore, the mRNAs expression of the factors involved in regulating muscle fiber growth of fish has great potential to optimize muscle quality. In fish, myogenic regulatory factors (MRFs) control the specification and differentiation of myogenic cells [21]. Myostatin 1 (MSTN1) and insulin-like growth factor 2 (IGF2) are the negative and positive regulators of muscle growth, respectively [22]. Paired box protein 7 (Pax7) can regulate the myogenic progenitor cell differentiation [23]. Previous studies investigated the influence of plant protein on muscle growth of fish. Alami-Durante et al. [24] found that plant protein increased the myoblast determination protein (MyoD) expression in rainbow trout, and Li et al. [25] found that faba bean meal improved the expression of MRFs in grass carp (*Ctenopharyngodon idellus*). However, research related to the effects of paper mulberry on muscle growth of fish is rare.

As a typical herbivorous fish, grass carp has a higher tolerance of plant sources compared to carnivorous and omnivorous fish. In addition, as one of the most productive freshwater fish in China, grass carp have huge yields and demands. The global production of grass carp in 2019 was more than 50 million tons [26]. With the improvement of living standards, consumers’ demands for high-quality aquatic products are increasing. Therefore, it has important economic significance to develop a plant raw material that can improve the muscle quality and even the growth performance of grass carp. The present study was conducted to investigate the influence of paper mulberry on the growth performance and muscle quality of grass carp.

## 2. Materials and Methods

This trial was performed in accordance with the Guide for the Care and Use of Laboratory Animals, and was approved by Hunan Normal University Institutional Animal Care and Use Committee (Changsha, China) under permit No. 20200012.

### 2.1. Experimental Diets

In the experiment diet, soybean meal (crude protein: 44%; crude lipid: 1.9%; energy: 14.26 MJ/kg) and fish meal (crude protein: 67%; crude lipid: 8.4%; energy: 13.47 MJ/kg) were used as the main protein sources, and soybean oil (crude lipid: 98%; energy: 36.6 MJ/kg) was used as the main lipid source. Five experiment diets were formulated by adding 0%, 5%, 10%, 15% and 20% paper mulberry (*Broussonetia papyrifera*, BP) into the commercial diet (crude protein: 31%, crude fat: 3.8%). The paper mulberry was obtained from Zhuozhou station of China Agriculture University (Hebei, China). The formulation of the diet is shown in Table 1. All ingredients were ground to pass a 40 mesh screen, mixed gradually and blended with soybean oil and water. Pellets with a diameter of 2 mm were produced by a laboratory pellet machine. After drying the pellets at 60 °C for 5 h, all diets were stored at −20 °C in plastic-lined bags until use.

### 2.2. Feeding Management

The experiment was carried out in the Chetianjiang Reservoir in Hunan (Xinghua, Hunan, China). The grass carps were brought from Xiangyin country (Yueyang, Hunan), and 600 grass carps were randomly divided into 15 floating cages (2.0 × 1.5 × 1.5 m) after acclimating for 1 week. There were three replicates for each dietary treatment, and each replicate contained 40 grass carps. Grass carps were fed at the rate of 3–4% of body weight 3 times per day at 8:00, 12:30 and 16:00 for 8 weeks. The feeding rate was adjusted on a weekly basis to feed the fish to apparent satiation. Water quality indicator data were as follows: water temperature was 26.0 ± 3.3 °C, pH was 7.3 ± 0.5 and dissolved oxygen was 7.0 ± 0.5 mg/L.

### 2.3. Sample Collection

At the end of the experiment, all fish were euthanized with MS222 (tricaine methane sulfonate, Sigma, St. Louis, MO, USA) at a concentration of 100 ppm after fasting for 24 h. All grass carps were counted and weighted for calculation of growth performance as follows:

Special gain rate (SGR) = (Ln (final body weight (g)) − Ln (initial body weight (g))/feeding days × 100

Feed conversion rate (FCR) = feed intake (g)/wet weight gain (g)

Survival rate (SR, %) = 100 × (final number of fish/initial number of fish)

A muscle of 1 cm × 0.5 cm × 0.5 cm from the right side of three carps per replicate was immersed in 4% paraformaldehyde for morphometric analysis, and the muscle on the other side (1 cm × 1 cm × 0.5 cm) was collected for texture analysis. A sample of approximately 9 g of fresh muscle from three carps (3 g per carp) per replicate was dissected, collected and mixed as one sample (*n =* 3 per dietary treatment) in a 10 mL microfuge tube, then stored at −20 °C for muscle nutritional component and quality analysis. A sample of approximately 3 g of fresh muscle from three carps (1 g per carp) per replicate was mixed as one sample (*n =* 3 per dietary treatment) in a 5 mL microfuge tube and stored at −80 °C for the determination of enzymatic activities and quantitative PCR.

### 2.4. Muscle Nutritional Component Analysis

The nutritional component analysis of muscle was conducted by standard methods (AOAC, 2005). Moisture was determined by oven drying to a constant weight at 105 °C in DHG—9240A (Keelrein Instrument Co., Ltd., Shanghai, China). Protein was determined by measuring nitrogen (N × 6.25) using the Kjeldahl method in FOSS Kjeltec 2300 (Foss Analytical Instruments Co., Ltd., Hillerød, Denmark). Lipid was determined by ether extraction using FOSS Soxtec 2050 (Foss Analytical Instruments Co., Ltd., Hillerød, Denmark).

### 2.5. Muscle Quality Analysis

The water-holding capacity (WHC) was conducted by the gravimetric method [27]. About 1 g of skinned muscle was weighed (S) and wrapped in the filter paper (V1), then put in a centrifuge tube and centrifuged at 4000× *g* for 10 min at room temperature. The wet filter paper (V2) was weighed and dried in an oven (75 °C) to constant weight (V3). The following formula was used to calculate the indicators: drip loss = 100 × (V2 − V3) × S^−1^; lipid loss = 100 × (V3 − V1) × S^−1^.

Muscle pH value was performed by a pH meter (testo-205, testo AG, Germany) at room temperature according to the methods described by a previous study [28].

The thiobarbituric reactive substances (TBARs) content was determined by the method of Wang et al. [29] with minor modifications. Muscle samples were homogenized in 20 mL of trichloroacetic acid solution (ratio of 1:5 (W/V)). After centrifugation at 3500× *g* and 4 °C for 15 min, the resulting supernatant was filtrated. Then, 5 mL of filtrate was mixed with 5 mL of 2-thiobarbituric acid (TBA) solution. At the same time, 5 mL of trichloroacetic acid solution and 5 mL of TBA solution were mixed as the blank. Both the sample solution and the blank were boiled in a water bath for 15 min. After being cooled with running water, the absorbance of the sample solution (As) and the blank (Ab) was measured at 530 nm. The TBA value (mg of malonaldehyde (MDA) per 100 g of tissue) was obtained by: TBA = 50 × (As − Ab)/200.

The total volatile base nitrogen (TVB-N) content was detected in FOSS Kjeltec 2300 (Foss Analytical Instruments Co., Ltd., Hillerød, Denmark) according to the GB/T 5009.228-2016. In brief, the standard titration solution of HCL (0.01 mol/L) was used for titration. The distillation time was set to 180 s, and the titration end point was set as pH = 4.65. At first, the reagent blank was measured with distilled water, adding 1 g of MgO. Then, the homogeneous solutions were composed of 10 g of fish muscle and 75 mL of distilled water with 1 g of MgO as the sample for detection. The determination was according to the conditions set and the requirements of the instrument operation manual. The TVB - N content (mg/100 g) was calculated as the following formula: TVB − *N =* ((V_1_ − V_2_) × C × 14/m) × 100.

V_1_: volume of HCl consumed by test solution (mL); V_2_: volume of HCl consumed by reagent blank (mL); C: concentration of HCl (mol/L); m: sample quality (g).

### 2.6. Muscle Texture Measurement

Muscle texture was evaluated using a TMS-PRO TPA device (Food Technology Corporation, Sterling, VA, USA) equipped with a flat-bottomed cylindrical probe (diameter: 8 cm) and a load cell of 250 N. The assay was performed according to the method described by Zhang [30]. All texture profile analysis (TPA) measurements were performed using 1 cm × 1 cm × 0.5 cm pieces from the dorsal white muscle of each fish. A test of double compression of the samples to 60% of their height was made. The speed of the roller movement during the test was 1 mm/s, the gap between pressures was 2 s. The TPA technique was intended to determine texture characteristics: hardness, adhesiveness, cohesiveness, springiness, gumminess and chewiness.

### 2.7. Muscle Enzymes Activity Measurement

About 1 g of muscle sample was homogenized in 9 mL of 0.9% ice-cold sodium chloride buffer and centrifuged at 3000× *g* at 4 °C for 10 min. The supernatant was collected for the contents of lactate, hydroxyproline (HYP), troponin T (TnT) and muscle glycogen analysis at the appropriate dilution. All of it was measured with a commercial kit provided by NanJing Jiancheng Bioengineering Institute.

### 2.8. Histological Analysis

For histological analysis, samples of muscle were fixed immediately in 10% buffered formalin, subsequently dehydrated with a series of ethanol, infiltrated in xylene and embedded in paraffin wax, as per standard histological protocols. A section of approximately 5 μm thickness was stained with hematoxylin and eosin (HE) and digitally photographed under a light microscope (BX40F4, Olympus, Tokyo, Japan).

For the analysis of fiber diameter, the muscle fiber was assumed to be cylindrical, and the diameter was calculated as s = πr^2^ (where s is the muscle fire area and r is the muscle fire radius), according to the method described by [31]. A size limit for identifying fibers was set at fiber diameter ≥ 10 µm, since the optical resolution below this limit did not allow for sufficient identification and accuracy in the analyses. Muscle fiber density = number of muscle fibers/area selected.

### 2.9. Real-Time Quantitative PCR Analysis

Total RNA was extracted using TRIzol reagent (Invitrogen, USA). The purity of total RNA was measured using a Nanodrop ND-2000 spectrophotometer (Thermo Fisher Scientific, Waltham, MA, USA) for the determination of OD_260_, OD_280_ and OD_230_ (OD260/280 ≥ 1.8 and OD260/230 ≥ 1.5). Subsequently, the total RNA was reversely transcribed into cDNA with Super-Script III RNase H-reverse transcriptase (Invitrogen, Carlsbad, CA, USA) according to the manufacturer’s instructions. The real-time PCR (q-PCR) was carried out in Bio-Rad CFX96 system (Redmond, WA, USA) with SYBR Premix Ex Taq Ⅱ (TaKaRa, Dalian, China). A total volume of 25 μL of the PCR reaction was composed of SYBR Premix Ex TaqⅡ (2×) 12.5 μL, forward primer 1 μL (10 μM), reverse primer 1 μL (10 μM), cDNA 2 μL (25 ng/μL) and sterile double-distilled water 8.5μL. The amplification protocol was as follows: pre-denaturation at 95 °C for 30 s, followed by 95 °C for 3 s and 60 °C for 25 s (40 cycles). The primers are listed in Table 2, and *β*-actin was selected as the internal control. The relative expression ratio (R) of mRNA was calculated using the following equation R = 2^−ΔΔCt^ [32,33].

### 2.10. Statistical Analysis

The statistical analyses were performed using software (SPSS 18.0). All data were presented as mean ± S.E. and analyzed by one-way ANOVA followed by Tukey’s test to inspect all differences among the dietary treatments. In addition, to determine if the effect was linear and/or quadratic, a follow-up trend analysis using orthogonal polynomial contrasts was performed [34]. A significant difference was considered at *p <* 0.05.

## 3. Results

### 3.1. Effect of Paper Mulberry on Growth Performance of Grass Carp

The effects of dietary paper mulberry on the growth performance of grass carp are shown in Table 3. Accordingly, BP had no significant influence on the SR of grass carp (*p >* 0.05). Increasing levels of paper mulberry linearly and quadratically decreased the SGR of grass carp (*p <* 0.05). In comparison to the control diet, when the supplementation of paper mulberry reached 10%, the SGR of grass carp decreased significantly (*p <* 0.05). Moreover, increasing levels of paper mulberry linearly and quadratically increased the FCR of grass carp (*p <* 0.05), and the FCR of the 15%BP and 20%BP groups were significantly higher than that of the control diet (*p <* 0.05).

### 3.2. Effect of Paper Mulberry on Muscle Nutritional Component of Grass Carp

As shown in Table 4, there were no significant differences in muscle moisture among all groups (*p >* 0.05). Positive quadratic trends were found between the paper mulberry levels and muscle crude fat or protein of grass carp (*p <* 0.05). In comparison to the control diet, the 10%BP and 15%BP groups had significantly decreased muscle crude fat and increased crude protein (*p <* 0.05).

### 3.3. Effect of Paper Mulberry on Muscle Texture of Grass Carp

**Figure 1:**
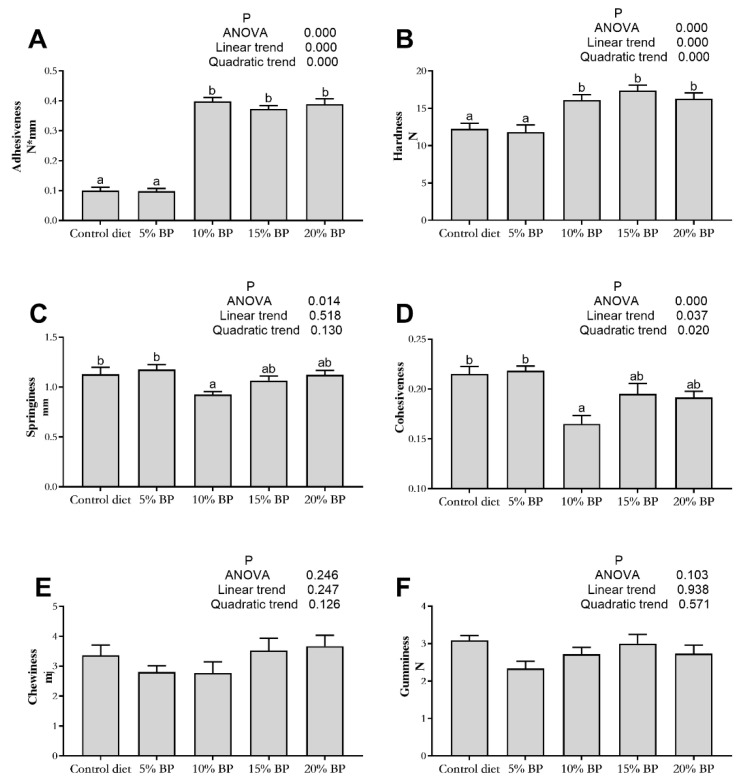
Effects of paper mulberry (BP) on muscle texture parameters of grass carp. (**A**) Adhesiveness; (**B**) hardness; (**C**) springiness; (**D**) cohesiveness; (**E**) chewiness; (**F**) gumminess. Different letters indicate significant differences (*p <* 0.05, *n =* 3).

As is described in Figure 1, no significant influence was observed on muscle gumminess and chewiness of grass carp (*p >* 0.05). The levels of paper mulberry linearly and quadratically influenced muscle adhesiveness, hardness and cohesiveness (*p <* 0.05). In comparison to the control diet, when the supplementation of paper mulberry reached 10%, the adhesiveness and hardness of grass carp increased significantly (*p <* 0.05). The cohesiveness in the 10%BP group was significantly lower than that of the control diet (*p <* 0.05).

### 3.4. Effect of Paper Mulberry on Muscle Water-Holding Capacity and pH of Grass Carp

The influence of paper mulberry on the WHC and pH of grass carp muscle are shown in Table 5. There were no significant differences in lipid loss and pH of grass carp muscle among all the groups (*p >* 0.05). Positive linear and quadratic trends were found between paper mulberry levels and muscle water loss of grass carp (*p <* 0.05). In comparison to the control diet, all the groups with the supplementation of paper mulberry had significantly increased muscle water loss of grass carp (*p <* 0.05).

### 3.5. Effect of Paper Mulberry on Muscle TVB-N and TBARs of Grass Carp

The influence of paper mulberry on the content of TVB-N and TBARs in grass carp muscle are listed in Table 6. Paper mulberry had no significant influence on TVB-N content in grass carp muscle (*p >* 0.05). The levels of paper mulberry significantly affected the muscle TBARs of grass carp (*p <* 0.05). However, no significant linear or quadratic trend was found between TBARs and paper mulberry contents. There was a significant difference between the 20%BP group and the control diet (*p <* 0.05).

### 3.6. Effect of Paper Mulberry on Partial Muscle Enzymes Activity of Grass Carp

As is described in Table 7, there are no significant differences in muscle lactate, HYP and TnT contents of grass carp muscle among all groups (*p >* 0.05). Positive linear and quadratic trends were found between paper mulberry levels and glycogen in grass carp muscle (*p <* 0.05), and the muscle glycogen of grass carp in the 20%BP group was significantly lower than that of the control diet (*p <* 0.05).

### 3.7. Effect of Paper Mulberry on Muscle Morphology of Grass Carp

**Figure 2:**
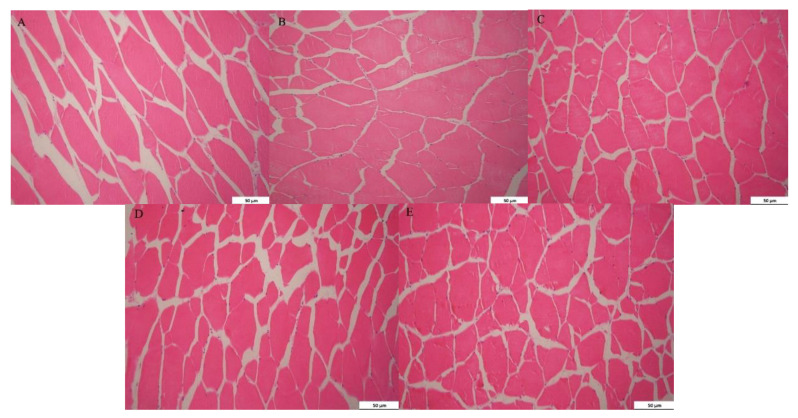
The morphology of the muscle of grass carp fed a diet with paper mulberry (BP) for 8 weeks. (**A**) Control diet 200×; (**B**) 5%BP group 200×; (**C**) 10%BP group 200×; (**D**) 15%BP group 200×; (**E**) 20%BP group 200×.

The results of HE staining are shown in Figure 2, and the effects of BP on muscle fiber diameter and density are listed in Table 8. The levels of paper mulberry linearly and quadratically affected the muscle fiber diameter and density of grass carp (*p <* 0.05). In comparison to the control diet, the muscle fiber diameter of grass carp decreased significantly in the 15%BP and 20%BP groups (*p >* 0.05). Moreover, the muscle fiber density of grass carp increased significantly when the addition of paper mulberry in the diet reached 10% (*p <* 0.05).

### 3.8. Effect of Paper Mulberry on Muscle Growth-Related Gene Expression of Grass Carp

**Figure 3:**
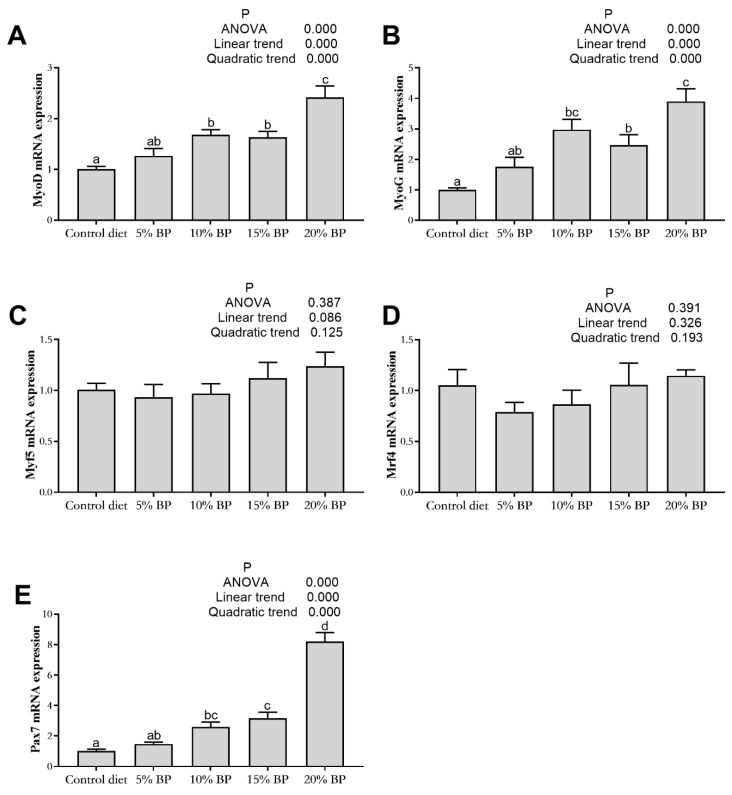
The gene expression of myogenic regulatory factors in the muscle of grass carp fed a diet with paper mulberry (BP) for 8 weeks. MyoD = myoblast determination protein (**A**), MyoG = myogenin (**B**), Myf5 = myogenic factor 5 (**C**), Mrf4 = myogenic regulatory factor 4 (**D**), Pax7 = paired box protein 7 (**E**). Different letters indicate significant (^a, b, c, d^) differences (*p <* 0.05, *n =* 3).

**Figure 4:**
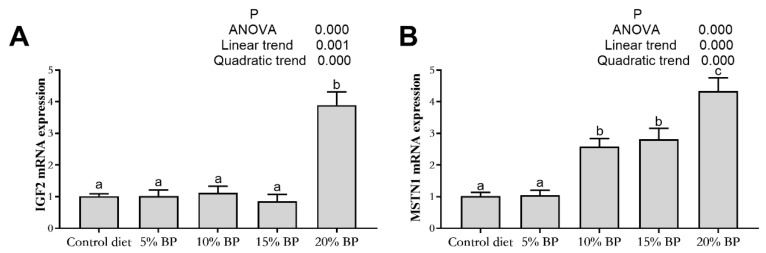
The gene expression of muscle growth regulatory factors in the muscle of grass carp fed a diet with paper mulberry (BP) for 8 weeks. IGF2 = insulin-like growth factor 2 (**A**), MSTN1 = myostatin 1 (**B**). Different letters (a,b)indicate significant differences (*p <* 0.05, *n =* 3).

As shown in Figure 3 and Figure 4, adding paper mulberry had no significant influence on myogenic factor 5 (Myf5) and myogenic regulatory factor 4 (Mrf4) expressions in the muscle of grass carp (*p* > 0.05). Increasing the dietary paper mulberry contents linearly and quadratically increased the expressions of MyoD, myogenin (MyoG), Pax7, MSTN1 and IGF2 (*p* < 0.05). In comparison to the control diet, the expressions of MyoD, MyoG, Pax7 and MSTN1 improved significantly after adding 10% paper mulberry to the diet (*p* < 0.05), and the expression of IGF2 was significantly increased in the 20%BP group (*p* < 0.05).

## 4. Discussion

The content of the crude protein of paper mulberry varies from 18% to 22%, which is higher than other xylophyta, such as *Morus alba* L. [35]. A previous study found that the lignin content and composition, especially the level of syringyl-lignin, affected the digestibility of forage [36]. Peng et al. [37] found that the caffeic acid 3-*O*-methyltransferase gene family that affected syringyl-lignin biosynthesis expended in paper mulberry through the genome analysis. Paper mulberry has been used to feed livestock for thousands of years, but few researchers have studied its effects on aquatic animals. Previous research found that adding 25% and 50% paper mulberry improved the growth performance of goat [11]. However, adding paper mulberry reduced the apparent digestibility of crude protein and dry matter in pigs, and the appropriate addition of paper mulberry in pigs was no more than 10% [8]. In the present study, adding 10% paper mulberry decreased the SGR of grass carp. These discrepancies may be due to the high fiber content of paper mulberry. In general, ruminant could digest the crude fiber effectively, but the high content of fiber affected the growth performance of monogastric animals. Compared with livestock, the digestive system of aquatic animals is more primitive. Research about fish found that high levels of fiber in the diet restricted the digestive enzymes function and affected the digestibility of Atlantic cod (*Gadus morhua* L.) [38] and rainbow trout (*Oncorhynchus mykiss*) [39]. Moreover, a previous study found that grass carps lack genes related to cellulase synthesis, even as an herbivorous fish [40]. Studies found that tannin decreased the feed intake of common carp [41], inhibited the activity of α-amylase and lipase and had toxicity on tilapia (*Oreochromis mossambicus*) [42].

The TBARs are wildly used as a biomarker of secondary lipid oxidation. Lipid oxidation damaged the meat quality through producing undesirable rancid off-flavors or even toxins [43]. In the present study, adding 20% paper mulberry significantly decreased the TBARs in the muscle of grass carp. It indicated that the diet with paper mulberry supplementation might be helpful to extend the storage time by preventing the lipid oxidation. This effect was due to the antioxidant function of paper mulberry. Preliminary studies proved that the polysaccharides extracted from paper mulberry had strong antioxidant activities [44,45], and a diet supplemented with 10% paper mulberry could improve the antioxidant capacity of cows [46]. However, in comparison to the cows, the TBARs in the muscle of grass carp decreased significantly only when the addition of paper mulberry reached 20% in our experiment. This may be because the primitive digestive system of grass carp limited the absorption of polysaccharides from paper mulberry.

Muscle pH and WHC are important parameters in evaluating muscle quality. After death, the muscle glycogen is degraded by glycolytic enzymes. It leads to muscle acidification and decreases the pH of the muscle [47]. In the present study, the content of muscle glycogen in the 20%BP group decreased significantly, but the content of lactate in the muscle was no different among all groups. Paper mulberry had no significant influence on muscle pH among all groups. This corresponded to the study of sheep [48] and pig [49]. The ability of meat to retain both inherent water and added water is defined as WHC [50], which has a great impact on the meat texture because the large reduction of water in meat leads to an increase in hardness [51]. The present study showed that adding paper mulberry increased the drip loss significantly. A similar result was found in the research of Song et al. [52], which found that adding paper mulberry decreased the WHC of pig muscle and the meat quality. Interestingly, Song et al. [52] found that in the same low-protein diet, the addition of fermented paper mulberry had no significant effect on the WHC of pig muscle. The influence of paper mulberry on the WHC of muscle might be related to the content of anti-nutrition factors in paper mulberry, because some studies proved that it was reduced after fermentation, especially tannin [16,53]. Besides WHC, the muscle fat content also affects the hardness of meat. Previous studies proved that the high content of fat affected the structure of muscle and decreased the firmness of meat [54,55]. In the present study, the muscle fat content decreased with the improvement of the addition of paper mulberry, and adding 10% and 15% paper mulberry had significant influence on it. This result showed that paper mulberry might affect the muscle texture of grass carp.

In order to further study the effects of paper mulberry on muscle quality of grass carp, muscle texture was detected by TPA. In this experiment, when the supplementation of paper mulberry reached 10%, the muscle hardness and adhesiveness of grass carp increased significantly. As mentioned above, the increase in hardness corresponded to the decline of muscle WHC and fat content in grass carp. Texture is one of the most important quality indicators of fish. Among textural parameters, muscle hardness is a vital factor affecting acceptability of fillets [56,57]. Previous studies found the feeding ingredients affected the textural qualities of grass carp. After feeding broad bean (*Vicia faba*), the muscle hardness of grass carp increased and the fillets of grass carp were more compact and crisper than ordinary grass carp [58,59]. In addition, soybean meal also increased the hardness of grass carp muscle [60]. However, Azm et al. [61] found that replacing the rapeseed meal with distiller’s dried grains with solubles negatively affected the hardness of grass carp muscle. In this experiment, the result that muscle hardness increased after adding 10% paper mulberry in the diet indicated that paper mulberry was helpful in improving the fillet quality of grass carp.

In this experiment, when the supplementation of paper mulberry reached 10%, the muscle fiber diameter of grass carp decreased while the muscle fiber density increased. Muscle hardness has been reported to be associated with the muscle fiber diameter and density. In general, the lower muscle fiber diameter resulted in the higher density of muscle fiber and higher muscle hardness [62]. Lin et al. [55] explained that this was because high fiber density represented a higher surface-to-volume ratio of muscle fibers and led to the higher amount of the connective tissue surrounding each fiber.

MyoD, MyoG, Myf5 and Mrf4 are the four members of MRFs. MyoD is critical in skeletal myogenesis due to its ability to initiate the myogenic program, and Myf5 exhibits redundant functions with myoD [63]. Moreover, both of these two factors were related to the proliferation of myoblasts [64]. MyoG and Mrf4 play essential roles during muscle differentiation [65,66]. Pax7 was proved to participate in the induction of the myogenic program during development through regulating MyoD or Myf5 expression [67]. In the present study, the expression of Pax7, MyoD and MyoG increased significantly after adding 15% paper mulberry. This indicated that paper mulberry would affect the muscle fiber development of grass carp. Similar results were reported in grass carp [68], Senegalese sole (*Solea senegalensis*) [69] and Zebrafish (*D. rerio*) [70] when fed with plant proteins. IGFs and MSTN are the positive and negative transcription factors of MRFs [71]. Previous research proved the negative influence of MSTNs on the muscle growth of grass carp [72], blunt snout bream (*Megalobrama amblycephala*) [71] and bighead carp (*Aristichthys nobilis*) [73]. In this experiment, the MSTN expression increased significantly after adding 10% paper mulberry. It was perhaps the cause of the decrease in growth performance.

## 5. Conclusions

With the increase in dietary paper mulberry content, the growth performance of grass carp decreased while the muscle quality of grass carp improved. When the supplementation of paper mulberry reached 10%, it had a negative effect on the growth performance of grass carp. However, it improved the grass carp muscle texture through improving muscle hardness and adhesiveness. Moreover, it reduced muscle fat accumulation and the muscle fiber diameter of grass carp.

## Figures and Tables

**Table 1:** Formulation and proximate composition of the experimental diets (dry weight, %).

Ingredient	Control Diet	5%BP	10%BP	15%BP	20%BP
Fish meal	2	2	2	2	2
Soybean meal	20	20	20	20	20
Rapeseed meal	30	30	30	30	30
DDGS	6	6	6	6	6
BP ^1^	0	5	10	15	20
Corn gluten meal	6	6	6	6	6
Wheat flour	31	25.3	19.5	13.7	8
Microcrystalline	0.26	1.25	2.34	3.44	4.43
Soybean Oil	2	1.71	1.42	1.12	0.83
Choline chloride	0.2	0.2	0.2	0.2	0.2
Calcium dihydrogen	1.5	1.5	1.5	1.5	1.5
Premix ^2^	1	1	1	1	1
Antioxidants	0.01	0.01	0.01	0.01	0.01
Ethoxyquin	0.03	0.03	0.03	0.03	0.03
Proximate analysis (%)					
Moisture	90.12	89.88	89.74	90.21	89.95
Crude protein	31.04	30.78	30.89	31.21	31.15
Crude lipid	3.88	3.98	3.92	3.75	3.77
Crude ash	8.67	8.88	8.82	8.56	8.75
Carbohydrate	35.46	35.05	34.86	34.36	33.95
Energy (MJ/kg)	14.96	14.79	14.83	14.75	14.68

BP = Paper mulberry (*Broussonetia papyrifera*). Paper mulberry was obtained from Zhuozhou station of China Agriculture University (Hebei, China). ^2^ Provided by MGOTer Bio-Tech Co., Ltd. (Qingdao, Shandong, China), mineral premix composition (per kg) as follows: KCl, 200 mg; KI (1%), 60 mg; CoCl_2_·6H_2_O (1%), 50 mg; CuSO_4_·5H_2_O, 30 mg; FeSO_4_·H_2_O, 400 mg; ZnSO_4_·H_2_O, 400 mg; MnSO_4_·H_2_O, 150 mg; Na_2_SeO_3_·5H_2_O (1%), 65 mg; MgSO_4_·H_2_O, 2000 mg; zeolite power, 3645.85 mg; VB1, 12 mg; riboflavin, 12 mg; VB6, 8 mg; VB12, 0.05 mg; VK3, 8 mg; inositol, 100 mg; pantothenic acid, 40 mg; niacin acid, 50 mg; folic acid, 5 mg; biotin, 0.8 mg; VA, 25 mg; VCP1, 5 mg; VE, 50 mg; VC, 100 mg; ethoxyquin, 150 mg; wheat meal, 2434.15 mg.

**Table 2:** Real-time PCR primer sequences.

Gene	Primer	Accession NO.
MyoD	F 5′-CCCTTGCTTCAACACCAACG-3′ R 5′-TCTCCTCTCCCTCATGGTGG-3′	GU218462
MyoG	F 5′-TGAGGGAGAGGAGACGACT-3′ R 5′-GCTCCAGAACAGGGTAGTAGT-3′	JQ793897
Mrf4	F: 5′-TCATTCAACTTGTGCCCTCC-3′ R: 5′-GCCCACTTTGCGATACCC-3′	KT899334
Myf5	F: 5′ -GGAGAGCCGCCACTATGA-3′ R: 5′ -GCAGTCAACCATGCTTTCAG-3′	AB012883
Pax7	F: 5′-CAAGAAACTGGCTCAATCCG-3′ R: 5′-TCGCAATCGTCCTCATCG-3′	KJ195463.1
IGF2	F: 5′-GCTTCCACAAACCGCCTACC-3′ R: 5′-AAAGAGTCTCCGCCGTTGCT-3′	EF062860
MSTN1	F: 5′-GCAGGAGTCACGTCT TGGCA-3′ R: 5′-GAGTCCCTCCGGATTCGCTT-3′	KM874826
*β* actin	F: 5′-GGCTGTGCTGTCCCTGTA-3′ R: 5′-GGGCATAACCCTCGTAGAT-3′	M25013

MyoD = myoblast determination protein, MyoG = myogenin, Mrf4 = myogenic regulatory factor 4, Myf5 = myogenic factor 5, Pax7 = paired box protein 7, IGF2 = insulin-like growth factor 2, MSTN1 = myostatin.

**Table 3:** Growth performance of grass carp fed a diet with paper mulberry (BP) for 8 weeks.

	Initial Weight (g)	Final Weight (g)	SGR (%)	FCR	SR (%)
Control diet	49.93 ± 0.07	141.42 ± 3.31 ^b^	1.71 ± 0.04 ^b^	1.46 ± 0.04 ^a^	95.00 ± 0.00
5%BP	50.20 ± 0.04	131.04 ± 1.97 ^ab^	1.57 ± 0.02 ^ab^	1.67 ± 0.03 ^ab^	90.00 ± 3.82
10%BP	50.00 ± 0.11	124.38 ± 5.47 ^a^	1.49 ± 0.07 ^a^	1.64 ± 0.08 ^ab^	92.50 ± 3.82
15%BP	50.03 ± 0.08	116.57 ± 3.77 ^a^	1.39 ± 0.05 ^a^	2.06 ± 0.04 ^c^	96.67 ± 2.20
20%BP	49.98 ± 0.04	121.25 ± 1.08 ^a^	1.45 ± 0.02 ^a^	1.84 ± 0.01 ^bc^	95.83 ± 0.83
*p* value					
ANOVA	0.156	0.004	0.004	0.000	0.421
Linear trend	0.783	0.001	0.001	0.001	0.341
Quadratic trend	0.398	0.000	0.000	0.003	0.399

SGR = special growth rate, FCR = feed conversion rate, SR = survival rate. Data are expressed as mean ± S. E. Values with different superscripts are significantly different (*p <* 0.05, *n =* 3).

**Table 4:** Muscle nutritional component of grass carp fed a diet with paper mulberry (BP) for 8 weeks.

	Moisture (%)	Crude Fat (% Dry Matter)	Crude Protein (% Dry Matter)
Control diet	78.42 ± 0.31	15.87 ± 0.44 ^b^	81.80 ± 0.16 ^a^
5%BP	78.15 ± 0.12	14.74 ± 0.19 ^ab^	82.67 ± 0.52 ^ab^
10%BP	77.81 ± 0.36	14.38 ± 0.22 ^a^	84.31 ± 0.26 ^c^
15%BP	78.47 ± 0.22	14.08 ± 0.32 ^a^	83.34 ± 0.33 ^bc^
20%BP	77.92 ± 0.48	14.92 ± 0.27 ^ab^	82.96 ± 0.20 ^abc^
*p* value			
ANOVA	0.538	0.016	0.003
Linear trend	0.510	0.064	0.093
Quadratic trend	0.763	0.002	0.003

Data are expressed as mean ± S. E. Values with different superscripts (^a, b, c^) are significantly different (*p <* 0.05, *n =* 3).

**Table 5:** Water-holding capacity and pH of muscle in grass carp fed a diet with paper mulberry (BP) for 8 weeks.

	Water Loss (%)	Lipid Loss (%)	pH
Control diet	5.28 ± 0.89 ^a^	2.64 ± 0.20	6.63 ± 0.05
5%BP	9.38 ± 0.44 ^b^	2.97 ± 0.21	6.81 ± 0.06
10%BP	10.56 ± 0.74 ^b^	2.71 ± 0.35	6.67 ± 0.04
15%BP	9.25 ± 1.02 ^b^	2.05 ± 0.30	6.73 ± 0.05
20%BP	8.77 ± 0.48 ^b^	2.66 ± 0.10	6.69 ± 0.01
P value			
ANOVA	0.000	0.139	0.113
Linear trend	0.028	0.293	0.858
Quadratic trend	0.000	0.568	0.524

Data are expressed as mean ± S. E. Values with different superscripts (^a, b, c^) are significantly different (*p <* 0.05, *n =* 3).

**Table 6:** The content of TVB-N and TBARs of muscle in grass carp fed a diet with paper mulberry (BP) for 8 weeks.

	TVB-N (mg/100g)	TBARs (mg MDA/kg)
Control diet	10.13 ± 0.10	0.45 ± 0.02 ^b^
5%BP	10.42 ± 0.50	0.32 ± 0.02 ^ab^
10%BP	10.28 ± 0.09	0.40 ± 0.01 ^ab^
15%BP	10.66 ± 0.13	0.42 ± 0.08 ^ab^
20%BP	11.08 ± 0.05	0.25 ± 0.00 ^a^
*p* value		
ANOVA	0.118	0.028
Linear trend	0.010	0.082
Quadratic trend	0.029	0.193

TVB-*N =* total volatile base nitrogen, TBARs = thiobarbituric reactive substances, MDA = malonaldehyde. Data are expressed as mean ± S.E. Values with different superscripts (^a, b^) are significantly different (*p <* 0.05, *n =* 3).

**Table 7:** Muscle enzyme activity in grass carp fed a diet with paper mulberry (BP) for 8 weeks.

	Lactate (mmol/gprot)	Hydroxyproline (ug/100mg)	Troponin T (ng/dL)	Glycogen (mg/g)
Control diet	2.59 ± 0.25	3.77 ± 0.25	387.22 ± 36.45	1.24 ± 0.05 ^b^
5%BP	2.33 ± 0.30	3.03 ± 0.19	428.94 ± 46.69	1.19 ± 0.03 ^ab^
10%BP	2.48 ± 0.29	3.46 ± 0.56	421.38 ± 28.75	1.26 ± 0.05 ^b^
15%BP	2.45 ± 0.15	3.41 ± 0.48	384.09 ± 45.39	1.15 ± 0.01 ^ab^
20%BP	2.36 ± 0.41	3.44 ± 0.55	472.58 ± 39.15	1.06 ± 0.02 ^a^
*p* value				
ANOVA	0.973	0.831	0.527	0.012
Linear trend	0.706	0.845	0.320	0.007
Quadratic trend	0.921	0.789	0.564	0.006

Data are expressed as mean ± S. E. Values with different superscripts (^a, b^) are significantly different (*p <* 0.05, *n =* 3).

**Table 8:** Muscle fiber diameter and density in grass carp fed a diet with paper mulberry (BP) for 8 weeks.

	Fiber Diameter (μm)	Fiber Density (N/mm^2^)
Control diet	62.31 ± 2.12 ^b^	108.30 ± 5.07 ^a^
5%BP	62.50 ± 1.30 ^b^	126.91 ± 7.66 ^ab^
10%BP	56.41 ± 1.76 ^ab^	139.67 ± 5.21 ^b^
15%BP	51.71 ± 0.66 ^a^	170.70 ± 5.73 ^c^
20%BP	51.26 ± 1.28 ^a^	185.05 ± 9.26 ^c^
*p* value		
ANOVA	0.000	0.000
Linear trend	0.000	0.000
Quadratic trend	0.000	0.000

Data are expressed as mean ± S.E. Values with different superscripts (^a, b, c^) are significantly different (*p* < 0.05, *n =* 3).

## Data Availability

The data presented in this study are available on request from the corresponding author.
